# Local pulses of electrical potential can induce long-range transient excitations in self-aligned molecular films

**DOI:** 10.1038/s41598-019-48836-4

**Published:** 2019-08-26

**Authors:** T. Dadalyan, T. Galstian

**Affiliations:** 1 Center for Optics, Photonics and Laser, Department of Physics, Engineering Physics and Optics, Université Laval. 2375 Rue de la Terrasse, Québec (Qc), G1V 0A6 Canada; 2 Yerevan State University, 1 Alek Manukyan St, Yerevan, 0025 Armenia

**Keywords:** Biomaterials, Membrane biophysics

## Abstract

Natural liquids can contain self-aligned molecules (such as liquid crystals and biological membranes) which give them unique properties of anisotropic diffusion, coupling between the molecular orientation and flow, etc. Here, we describe the observation of new phenomena in those materials: long-distance transport and molecular orientation waves that are induced by pulses of spatially localized electrical potential. As a result, the morphological properties of the material are significantly altered well beyond the reach of the electrical field. The local dielectric torque-induced reduction of the effective molecular volume and corresponding pressure gradients are in the origin of these phenomena. Our observations are made for electric fields that are an order of magnitude smaller than those present in biological membranes. Thus, this discovery may have important impact on the understanding of the operation of these membranes and on the dynamics of action potential propagation in neural cells. The corresponding possible influence of observed excitation mechanisms on the ionic gates and the role of myelin sheath are discussed.

## Introduction

Liquid crystalline (LC) materials are typically composed of asymmetric (cigar or disk shaped) molecules whose orientation is correlated with their neighborhood at long distances, while their positional order is negligible, as in ordinary isotropic liquids^1^. This correlation is in the origin of several extraordinary phenomena, including the anisotropic^1^ and “elastic”^2^ diffusion of molecules, the extreme sensitivity of the molecular orientation to the material flow^1,3,4^ and to external stimuli such as magnetic or electric fields^1,5,6^. These materials have been intensively studied^1,5,7^ and used in light modulators and displays^8,9^, but their similarity with biological tissue (such as cell membranes and myelin^10^, mucus^11^, synovial fluid^12^ and biofilm^13^) has generated significant renewed interest in the scientific community^14–18^, including the study of microorganisms^19–24^ and of drug delivery processes^2,25^. While the anisotropic diffusion of molecules and ions is present in all above-mentioned tissue-examples, cell membranes represent a particular interest because of local pulses of electrical potential generated during the action potential’s propagation^26^. As we shall show below, such pulses may significantly modify the anisotropic properties of membranes.

## Theory

In an isotropic liquid, the effective distance *d* between the centers of masses of its molecules is defined by their electronic properties (e.g., dipolar) as well as by the magnitude of thermal Brownian motion. The situation should be different in a LC because of the anisotropy of its molecules^1^. Namely, the orientational fluctuations of these molecules must also be taken into account when estimating the value of *d*. Consequently, the effective volume *v*, occupied by those molecules, must be influenced by the magnitude of their orientational fluctuations too. This is important because of the extreme sensitivity of those fluctuations to various external stimuli^1^ (including electric fields), while in previous descriptions of the response of LCs to those stimuli, the density and the volume of LCs were considered as constant (approximated as an *incrompressible liquid*)^1,3^. In reality, it was discovered that those molecular fluctuations may be significantly affected not only by temperature variations (as is well-known), but also by an external electric field ***E***, at fixed temperature^27^. A legitimate question is then raised: *what is the influence of the electric potential on the density of such self-aligned molecular films*?

**Figure 1:**
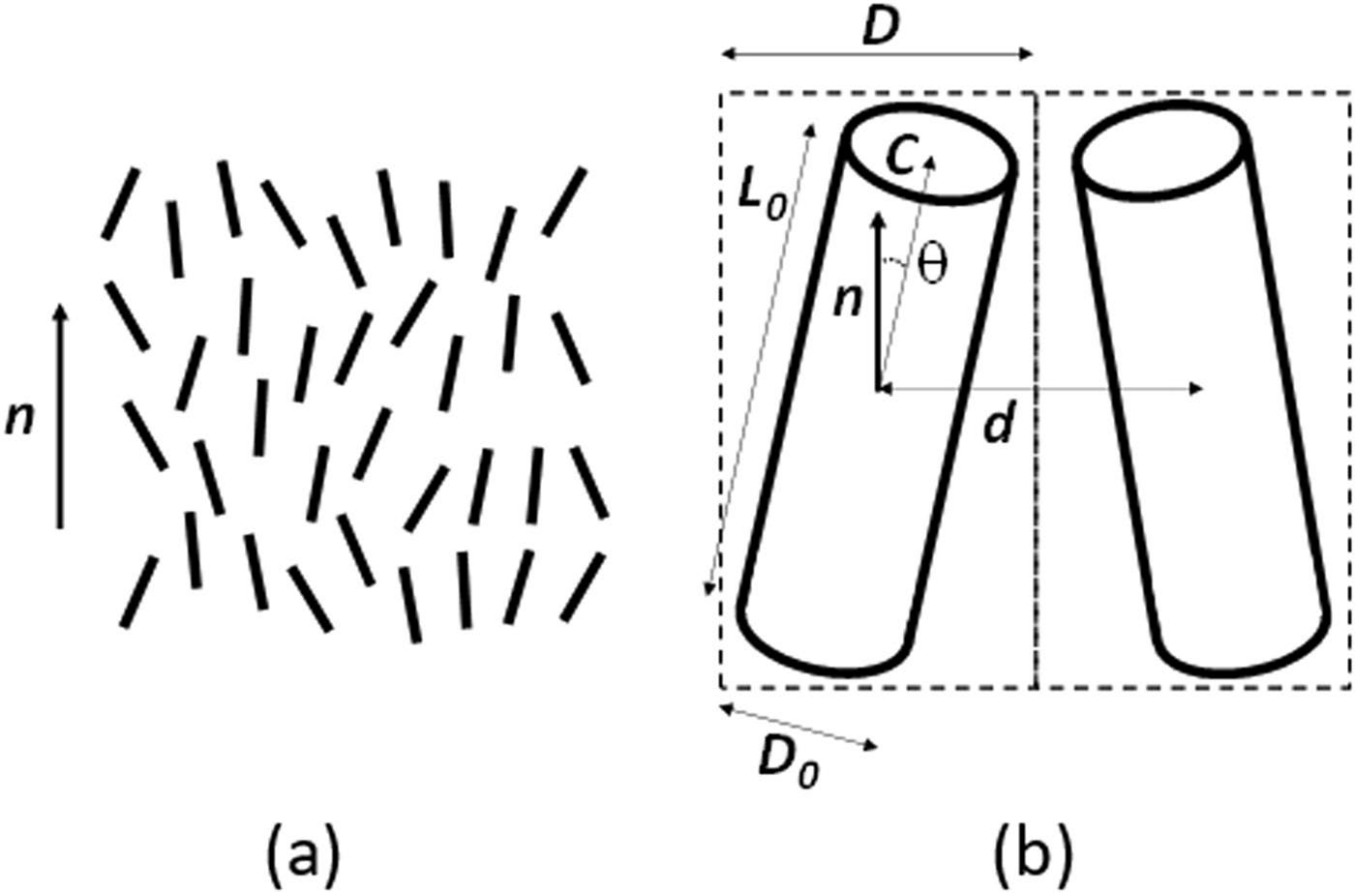
Schematic representation (**a**) of a nematic liquid crystal (short bars representing individual molecules) and of its director ***n***; (**b**) of the effective molecular volumes (dashed rectangles) occupied by two molecules (two tilted cylinders).

We predict that an electric potential reduces the effective volume of those films by reducing the thermal fluctuations of its molecular orientation. To prove this hypothesis and to analyze its consequences, we shall further consider nematic LCs (NLCs)^1^, which are uniaxial liquids with an anisotropy axis defined by the local average molecular orientation (represented by a unit vector ***n***, called *director*), Fig. 1a. To simplify our analyses, we shall use the model of rods or cylinders (Fig. 1) to describe the behavior of individual molecules. Thus, the diameter *D*_0_ of each cylinder (Fig. 1b) will correspond to the effective distance of neighboring molecules in the case of perfect parallel alignment. Obviously, the effective volume, occupied by the molecule, will then be *v*_0_ = *L*_0_π(*D*_0_/2)^2^, where *L*_0_ is its effective length.

The orientational distribution of those cylinders may be described by the angle θ between their axis *C* and the local director ***n*** (Fig. 1b). In NLCs, the level of orientational correlation is described by the order parameter *S*^1^ that is defined as

1 $$S=0.5 < 3\,{\cos }^{2}{\rm{\theta }}-1 > $$

The value of *S* is typically between 0.3 and 0.7, while *S* = 1 in a perfectly ordered NLC (with θ = 0) and *S* = 0 in an isotropic liquid (when the orientational order is completely lost).

At the same time, it is well known that NLCs are very sensitive to external electric fields ***E***^5^ that exert a dielectric torque **Γ** on their director:

2 $$\Gamma =({{\rm{\varepsilon }}}_{a}/4{\rm{\pi }})\langle ({\boldsymbol{nE}})({\boldsymbol{n}}\times {\boldsymbol{E}})\rangle $$

where ε_a_ ≡ ε|| − ε_⊥_ is the dielectric anisotropy of the NLC (ε***||*** and ε_⊥_ being the dielectric constants in the directions parallel and perpendicular to the director ***n***, respectively). This torque vanishes (**Γ** = 0) for two extreme cases of electric field orientation: when it is parallel (***n||E***) and perpendicular (***n*** ⊥ ***E***) to ***n***. Most importantly, no matter how strong the field, there is no reorientation of ***n*** if the NLC has a positive dielectric anisotropy (ε_a_ > 0) and the field is parallel to the director, ***n||E***.

However, the above mentioned torque is usually described in the framework of the “collective” interaction of molecules with the field ***E***^1^, which is much stronger than the interaction of ***E*** with individual molecules (∝ ***E*** or ∝ ***E***^***2***^, depending on whether they are related to their permanent or induced dipole moments). Despite this fact, it was reported that the value of S is significantly increased by means of an electric field ***E***^27^:

3 $${\rm{\bigtriangleup }}S(E)=a{\boldsymbol{E}}+b{{\boldsymbol{E}}}^{2}+\ldots $$

(where *α* and *β* are the coefficients of linear and quadratic dependences) and this field dependence of *S* was even used to obtain nanosecond electro optic switches in NLCs *without* reorientation of the director^28^. This shows that the torque, exerted on quasi individual molecules (individual molecules that are thermally fluctuating around the director), can be important. However, the spatial (long-range) impact of such orientational interactions was not analyzed in these works since the NLC samples were excited uniformly. To evaluate this impact, we can consider a situation when the NLC is far below the nematic-isotropic phase transition temperature T_N-I_. In this case, we have a well oriented material (with small angular deviations θ) and the order parameter *S* can be approximated as

4 $$S\approx 1-3/2 < {\theta }^{2} > ={S}_{0}+{\rm{\bigtriangleup }}S(E)$$

Furthermore, for moderate values of ***E***, the dependence (3) can be approximated as linear, providing a linear dependence between the field ***E*** and the angle δ (where δ is the field induced small reduction of the original angle θ_0_, that is, θ = θ_0_ − δ).

5 $$ < {\theta }_{0}\delta  > \approx a^{\prime} {\boldsymbol{E}}$$

**Figure 2:**
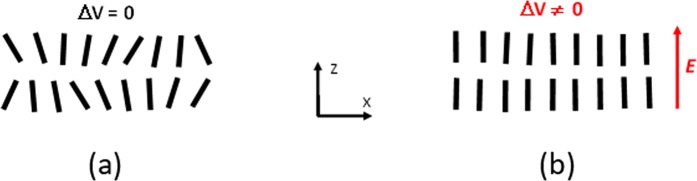
Schematic demonstration of the order parameter increase (in **b**) when an electric field *E* (here vertical) is applied to the orientationally correlated molecular layer (in **a**).

Thus, the higher the field ***E***, the lower the value of molecular fluctuation angle θ (Fig. 2), this being an *isothermal* process in contrast with the traditional way of increasing *S* by reducing the temperature of the material^29^. In this case, the effective molecular volume *v* will be reduced for higher values of ***E*** (Fig. 1b). The ratio of volumes *R* = *v/v*_0_ for different values of θ can be approximated as

6 $$v/{v}_{0}\approx 1+2({L}_{0}/{D}_{0}){\rm{\theta }}$$

Thus, the predicted volume change should depend upon the electric field ∝ ***E*** and this dependence should be higher for NLC molecules with higher aspect ratio *L*_0_/*D*_0_. Consequently, the macroscopic volume V of the material also should shrink (at least in the lateral × dimension, Fig. 2) in the presence of such a stabilizing electric field. It follows that a spatially non-uniform electric field ***E*** will generate local volume changes, which will result in the formation of pressure gradients.

## Results

### Experiment

**Figure 3:**
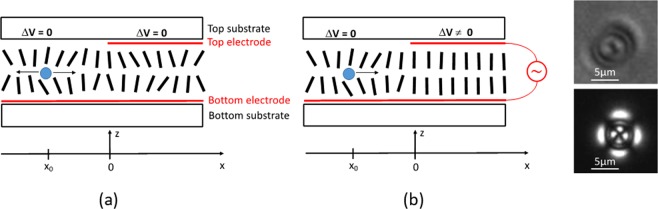
Schematic cross-section representation of (**a**) the ground state LC cell with a uniform ITO electrode on the bottom substrate and a non-uniform ITO electrode on the top substrate; (**b**) the LC cell with an electric potential applied to its right half area. *Right inset pictures*: microphotography of a microparticle with (bottom) and without (top) crossed polarizers. The white horizontal bar measures 5 μm.

To experimentally verify this hypothesis, we have fabricated a cell containing a homeotropically aligned NLC layer (LC molecules are perpendicular to two substrates of the cell). Two glass plates of 0.55 mm thickness and of approximately 16 mm × 16 mm lateral sizes were used as top and bottom substrates to build the LC cell (Fig. 3a). The bottom substrate was covered by a uniform transparent electrode (made of indium tin oxide, ITO), followed by a thin layer of Polyimide (PI5661, Nissan chemicals, not shown in the figure for the sake of simplicity) that caused a perpendicular orientation of LC molecules. The top substrate was similar to the bottom substrate with one important difference: only the right half of the substrate’s surface (x > 0) was covered by the ITO (Fig. 3a). Thus, the “right” area of the LC cell had two ITO layers facing each other, while the “left” area of the LC cell (x < 0) had only one ITO layer (this allows the application of spatially non-uniform electric potential, see below). The distance between two substrates was maintained by spacers (diameter = 20 μm) inserted into peripheral adhesive walls. These walls were used to contain the NLC material (5CB from Aldrich) inside the cell. In addition, borosilicate microspheres of 5 μm diameter (from Duke Chemicals, schematically shown by the filled circle in the left area of the LC cell, Fig. 3) were introduced into the cell in the process of filling it, using capillary action, with NLC material. These microparticles were used as received (no special treatment was applied to their surface). After filling the NLC material, the cell was heated above the temperature T_N-I_ (35.1 °C^30^,) and cooled down to room temperature (20 °C) to obtain a stable material system in the ground state (without an electric potential being applied). A good uniform alignment of NLC was obtained in the entire LC cell except in the immediate vicinity of microparticles (see the right inset of Fig. 3).

We used polarizing microscopy and polarimetry techniques (see hereafter) to study the behavior of the obtained NLC film during and after applying the electric potential to the right-half area of the cell.

**Figure 4:**
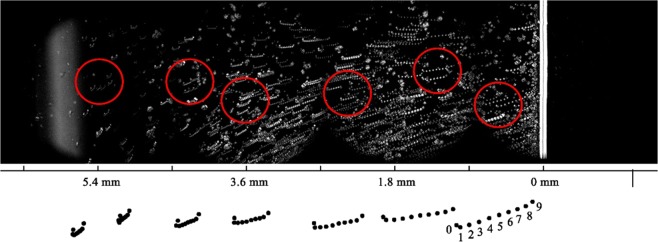
Combination of microphotographs (recorded with 3 minutes interval during 30 minutes of applied electric potential) demonstrating the movement of microparticles. White dots on the photograph correspond to sequential positions of microparticles. The dark rectangle on the right side (x > 0) is the area where the electric potential is applied between two ITO layers. The bright vertical zone is the limit of the top ITO. Black dots below the photograph schematically demonstrate zoomed trajectories of certain particles taken from the areas marked by six large circles.

Our microscopy observations showed that the application of the square shaped AC signal (with amplitudes ≥30 V_RMS_) generates important movement of microparticles (with 5 μm diameter) from the left area of the LC cell (where there is no ***E*** field) in the direction of +x, that is, towards the line separating the right and left areas of the LC cell (the limit of the ITO layer on the top substrate, at x = 0, Fig. 3). It is worth noticing that the electric field-induced transport of microparticles in NLCs was already studied^31,32^. However, this was done for particles, which were in areas directly exposed to the electric field (here, for x ≥ 0, Fig. 3). In our case, even particles that are in the far left area (x < 0, Fig. 3), moved towards x = 0. This movement is summarized in Fig. 4 (for a switched ON and maintained electric potential) by combining multiple microphotographs, periodically captured during 30 minutes (the LC cell was placed between crossed polarizers of the microscope, the top ITO limit line being oriented along the diagonal). The black dots on the bottom of the Fig. 4 correspond to the zoomed trajectories of some of those microparticles having different initial positions with respect to the top ITO limit line (Fig. 3). Large circles (Fig. 4) identify the approximate positions of these microparticles. The numbering on one of the trajectories (on the bottom right) shows the sequential positions of the particle with an interval of 3 minutes (the numbering on all other trajectories is similar). The electric field was switched ON immediately after capturing the position of the microparticle numbered 0 and was switched OFF after recording the position 8. The dark rectangle on the right side of the Fig. 4 corresponds to the area where the electric potential is applied (the area x > 0 in the Fig. 3). The bright vertical double-lane (at x ≈ 0) is the limit line of the top ITO. This increased light transmission is generated due to the reorientation of NLC molecules under the influence of the so-called *fringing* field (transition zone at x ≈ 0 mm, where there is a decreasing tilted ***E*** field).

**Figure 5:**
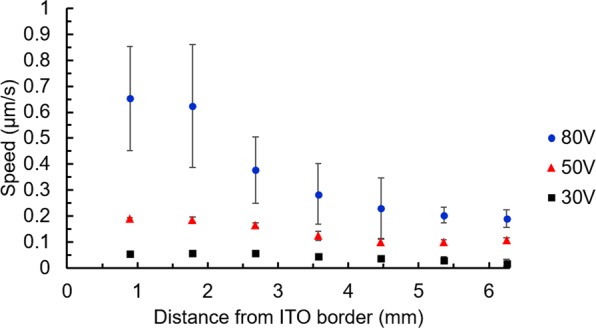
Recorded speed of movement of microparticles depending upon their original distance from the top ITO limit line (x = 0) when various voltages were applied on electrodes (squares: 30V_RMS_, triangles: 50V_RMS_ and circles: 80V_RMS_, all at 1 kHz).

As we can see (Fig. 4), these microparticles are far away from the area where the electric field is applied. Their movement continued for as long as the potential was maintained (up to 30 minutes). As we can see from the summary of the Fig. 5, the higher the applied potential and the closer (originally) the microparticles to the position x = 0, the stronger (in speed and distance) their movement. If we applied the potential long enough, the movement continued until the microparticles reached the top ITO’s limit line and were trapped in a circular movement (to be described in detail elsewhere).

The direction of movement was not perfectly linear (Fig. 4): just after switching ON the potential, the microparticles underwent a small side-jump (e.g., in the direction of +y), that is, parallel to the top ITO’s limit line. Then the microparticles moved towards the +x direction with slight tilt angle (depending upon their original position).

Upon the removal (switching OFF) of the potential, the microparticles underwent a jump in the opposite direction (in the direction of −y) and then started moving back (in the direction of −x). However, this movement was rather quickly attenuated (after few minutes) and the microparticles did not return to their original positions if the excitation period was long enough.

**Figure 6:**
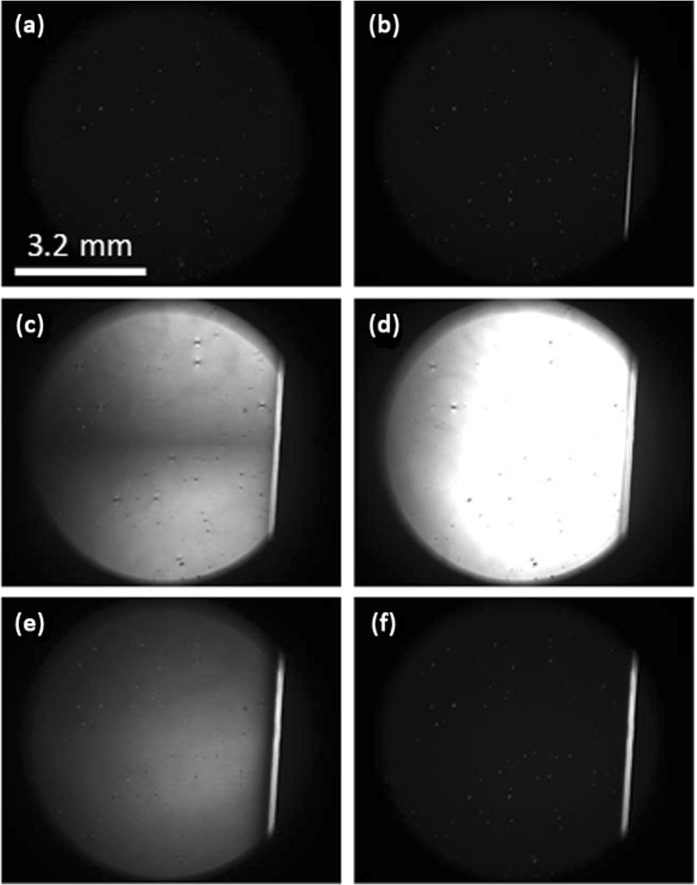
Polarizing microscope photographs of the LC cell in the (**a**) ground state (before the electric potential is applied), and when an electric potential of 80V_RMS_ is applied (to the right area of the LC cell) and kept constant, (**b**) potential is switched ON (the fringing field area can be identified by the vertical bright line on the right), (**c**) 40 msec later, (**d**) 80 msec later, (**e**) 220 msec later, (**f**) 340 msec later.

Further polarizing microscopy observations of the early stages of excitation (the LC cell was always placed between two cross oriented polarizers with the ITO’s limit-line aligned by the diagonal; the microscope’s probe light incidence on the cell was normal) showed that the application of the electric potential provoked another interesting phenomenon. Beforehand, in the ground state of the LC cell, some small bright spots (due to orientational defects around microparticles or their aggregates, see the inset of Fig. 3) could be seen across the entire LC cell (Fig. 6a). The abrupt application of the electric potential to the right side of the LC cell resulted into a strong transient reorientation of the director at its left area generating increased light transmission (bright zone on the left, Fig. 6c–e). The transmitted light intensity oscillated and then returned almost to its original level Fig. 6f (to be compared with Fig. 6a) at a constantly maintained electric potential.

This reorientation wave was very fast (compared to the movement of microparticles described above) and propagated for very long distances: it reached the left limit of the LC cell (at x ≈ −6 mm) within ≈0.1 sec. Thus, the director reorientation was as far reaching (non-local in space) as the microparticles’ movement, but it decreased quickly (within ≈0.36 sec), while the microparticles’ movement continued during the entire time the electric potential was applied (up to 30 minutes).

During the excitation process, there was no director reorientation on the right area of the LC cell (see at the right area of the bright vertical line in Fig. 6b–f) for the simple reason that there was a strong stabilizing electric field (see also the right  areas of Figs 3b and 4). In fact, the LC cell becomes darker (on the right area, Fig. 6b) compared to the ground state (before excitation). Given the geometry of the experiment (light propagates in the direction of average molecular orientation and the LC cell is placed between two cross oriented polarizers), it is clear that we observed an increased orientational order *S*.

**Figure 7:**
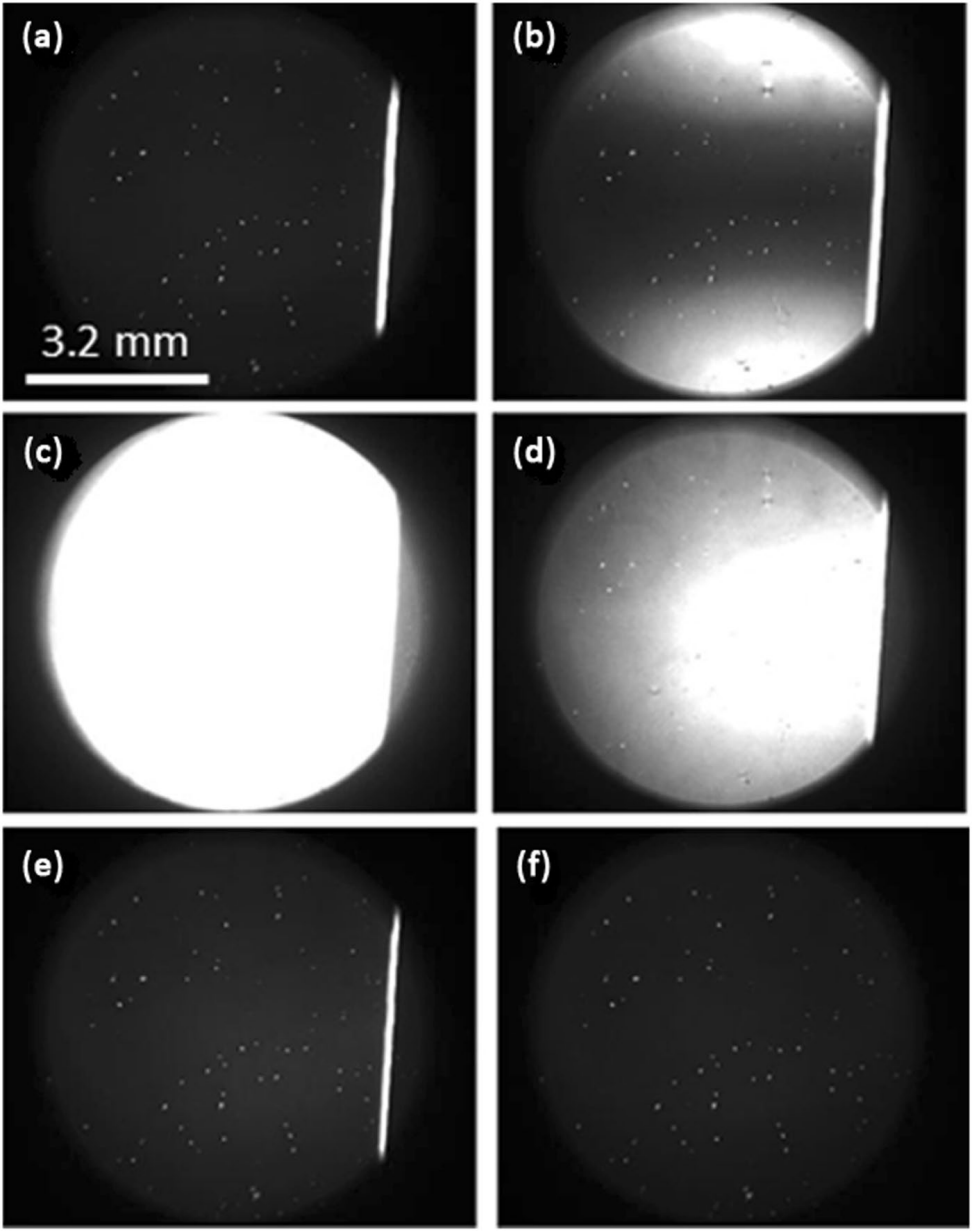
Polarizing microscope photographs of the LC cell (**a**) in the established excited state when still the same potential (80V_RMS_) is applied as in Fig. 6 (photo exposure is slightly different), and during the relaxation, when the electric potential is abruptly removed (**b**) just after the potential is switched OFF, (**c**) 40 msec later, (**d**) 180 msec later, (**e**) 300 msec later, (**f**) 1900 msec later.

The behavior of the NLC film was also very interesting when the voltage was abruptly switched OFF (Fig. 7). Namely, another fast wave of director reorientation was generated,

which, this time, penetrated into the right area of the LC cell as well (there was no more stabilizing electric field applied in that area). The reorientation dynamics (during the relaxation) were quite similar to those in the case of excitation (Fig. 7c–e). Finally, the LC cell almost returned back to its original state after the fast orientational wave disappeared (compare Fig. 7f with Fig. 6a).

**Figure 8:**
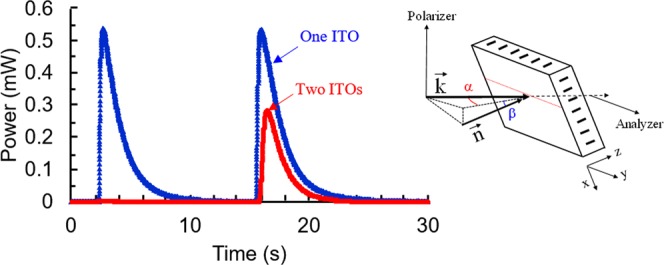
Transmitted power (mW) dependence upon time (in sec) for a 45° tilted LC cell, detected at x ≈ −2 mm (blue triangles) and at x ≈ 2 mm (red circles). The electric potential (80V_RMS_) was abruptly switched ON approximately at 2.3 sec and then abruptly switched OFF approximately at 15.6 sec. *Inset:* geometrical conditions of the experiment. ***k*** is the probe beam’s wavevector. The *xyz* axes are attached to the LC cell.

We performed some additional quantitative experiments to further investigate the character of the orientational perturbation of the NLC by using a macroscopic polarimetric set-up. The LC cell was placed (see the right inset of Fig. 8) between cross oriented polarizer (with vertical transmission axis) and analyzer (with horizontal transmission axis). The probe beam (CW He-Ne laser, operating at 632.8 nm) of 0.5 mm diameter was incident (with a wavevector ***k***) on different areas of the LC cell (always far from the top ITO limit line to avoid the contribution of the fringing field). As a *starting point*, the wavevector ***k*** of the probe beam, the ground-state director ***n*** and the ITO limit line (red dotted line, parallel to y axis) were all aligned in the horizontal plane, with vectors ***n*** and ***k*** being parallel, that is, ***n***’s orientation angles α = 0 (in the horizontal plane) and β = 0 (out of the horizontal plane).

For our first polarimetric experiment, the LC cell was first rotated (from the starting point) around the top ITO limit line to have its ***n*** oriented at β = 45° (out of horizontal plane) and then the cell was additionally rotated at α = 40° around the vertical axis (the top ITO limit line remains in the horizontal plane). In this configuration, there is already light transmission (through the analyzer) in the ground state since the incident probe beam generates both ordinary and extraordinary polarization modes in the LC cell and they have different effective phase evolution speeds. When we excite the medium, the reorientation of the director changes the effective birefringence Δn_eff_ of the medium and, consequently, the optical phase difference *ϕ* = d_eff_ Δn_eff_ 2π/λ (where λ is the wavelength of the probe and d_eff_ is the effective thickness of the LC layer) between these two polarization modes. Then the intensity of the transmitted (through the analyzer) light oscillates I ∝ sin^2^(*ϕ*/2) every time when this change is multiple of 2π. We have observed more than 1.5 such oscillations of transmitted light power in the part of the LC cell having only one ITO layer, both upon the switching ON and switching OFF the electric potential. This simply confirmed the propagation of the reorientation wave (and its level, see below) across the left area of the LC cell that we already observed by microscope.

Most interestingly however was the second polarimetric experiment when the cell was only rotated (again, from the starting point) by β = 45° around the y axis, while we kept α = 0 (so the top ITO limit line remains in the horizontal plane, but the ground state director ***n*** is now pointing out of that plane). In this case, only an extra ordinary polarized mode (in the plane xz) is generated in the ground state and the transmission (through the analyzer) must be minimal (it is indeed the case, see the initial signal in Fig. 8). This situation must remain unchanged if there is a director reorientation, which is in the same plane xz. The only possibility for the probe beam to traverse the analyzer is if the director’s reorientation is out of the plane xz (to generate two polarization components of the probe beam inside the LC cell). The corresponding results obtained (for 80V_RMS_) are shown in Fig. 8 for the abrupt switching ON (approximately at 2.3 sec) and then abrupt switching OFF (approximately at 15.6 sec) of the electric potential. Blue triangles represent the behavior for the probe beam traversing the area of the LC cell with only one ITO layer. As we can see, there is indeed a strong molecular “out-of-plane” reorientation with a characteristic time of ≈0.44 sec. Similar reorientation is present (in the same area of the LC cell) upon the removal of the electric potential (so, during the relaxation process). At the same time, on the other part of the LC cell (with two ITO layers), the reorientation is observed only during the relaxation process (red circles, Fig. 8). These observations are well correlated with our observations made using the polarizing microscope (Figs 6 and 7). In both cases (excitation and relaxation) the reorientation on the single-ITO area of the LC cell had almost the same amplitude and duration. The relaxation time of

7 $${\tau }_{{\rm{r}}}\approx \gamma {{\rm{L}}}^{2}/({{\rm{K}}}_{1}{{\rm{\pi }}}^{2})\approx 0.56\,\sec $$

that was theoretically estimated (for strong boundary conditions θ(0) = θ(L) = 0)^5,33^, and by using the orientational viscosity γ ≈ 0.096.8 Pa s^34^, NLC thickness L = 20 μm and splay elastic constant K_1_ ≈ 7pN^35^) was close to the experimentally observed values.

## Discussion

The fact that, upon the application of the electrical potential, microparticles are moving towards the top ITO’s limit line (in the direction of +x) shows that the observed phenomenon is not related to the dielectric heating^36^. A very small heating (ΔT < 1 °C, for which the density change is small^37^) was indeed observed in our experiments (performed with *f* = 1 kHz electric signal), but it became significant (ΔT ≈ 10 °C) only for very high frequencies (*f* > 10 kHz). In fact, we have an electric field only in the area x > 0 (with two ITO electrodes facing each other) and this part may be heated by this field because of the dielectric losses of the LC material. In this case, the LC would expand in that area (x > 0), which would apply pressure towards the zone x < 0. Thus, the thermal expansion of the NLC (due to the possible dielectric heating in the area x > 0) should push the NLC material and microparticles towards −x (away from the ITO limit line), which is not the case here.

Similarly, the particle movement cannot be initiated by the possible electrical attraction of two substrates and the resulting reduction of the NLC gap in the area x > 0^38^, since, in this case also, the movement of NLC material (and of microparticles) would be in the −x direction.

Finally, the observed movement cannot be explained by direct electric field induced forces either^31,39^ since those perturbations are observed far away (up to 6 mm) from the area with electrical potential.

In contrast, the well-known back-flow phenomena (flow induced by the director reorientation^1,6^) could contribute, but only at the very early stages of the process. Indeed, in our case ε_a_ > 0 and the electric field was parallel to the ground state director everywhere except in the zone x ≈ 0 (due to the fringing field). The non-uniform reorientation of the director in that zone will generate a material back-flow, but the characteristic time of this flow

8 $${\tau }_{{\rm{bf}}}\approx {{\rm{\rho }}{\rm{L}}}^{2}/({{\rm{\pi }}}^{2}{{\rm{\eta }}}_{1,2})\approx 5.8\times {10}^{-7}\,\sec $$

(where ρ ≈ 1.008 g/mL is the density of the NLC and η_1,2_ ≈ 20–120 cP = 0.02–0.12 Pa⋅s^40^, are the viscosity coefficients) is much shorter than the director’s orientation (excitation and relaxation) times τ_r_ (eq. 7). Thus, the back-flow effect will contribute only at the very beginning of the excitation (when the electric potential is just switched on). As we have seen (Figs 6 and 7), the reorientation in the fringing field area (the bright vertical line at x ≈ 0) was indeed started much faster (and remained stable) compared to the orientational wave propagation (seconds) and the microparticle transport (minutes).

Our basic hypothesis (about the excitation mechanism) was based on the effective molecular volume reduction by the application of an electrical potential. Obviously, the higher the temperature, the higher the ground-state molecular fluctuations (the value of θ is higher and, consequently, the value of *S* is lower) and the higher the effective volume of NLC molecules. Thus, the electric potential induced volume reduction, described in our work, is sensitive to the temperature of the film (those changes must be more pronounced for higher temperatures).

**Figure 9:**
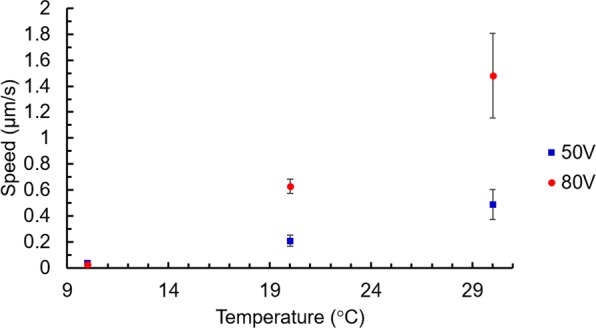
Dependence of the speed of microparticles upon the temperature of the LC cell for two values of electric potential (squares: 50V_RMS_ and circles: 80V_RMS_). The speed of particles was measured at ≈1.5 mm distance from the top ITO limit line.

We have performed such “control” experiments at various temperatures (below the T_N-I_ and by using the same procedures as described above). The results we obtained (Fig. 9) confirm this prediction (Instec thermal stage was used to control the temperature of the LC cell). In fact, as we can see, the observed dependence of the speed of the microparticles’ movement upon temperature is rather strong (for a 10 °C increase of temperature the speed of microparticles is more than doubled).

**Figure 10:**
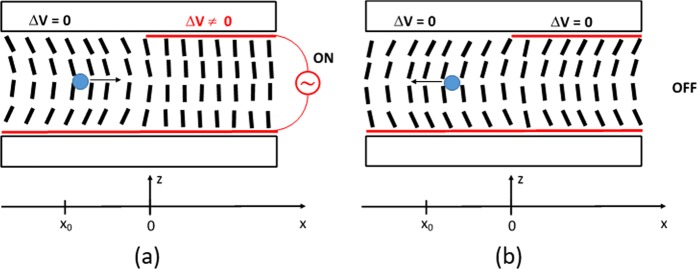
Schematic cross section demonstration of (**a**) the formation of the pressure gradient, orientation wave towards −x direction and particle movement towards +x direction when the potential is switched ON and (**b**) the relaxation process (when the potential is switched OFF) that penetrates into the +x area too.

Based on all above mentioned observations, we think that the main mechanism of excitation here is indeed related to the spatially local ***E***-field induced reduction of the effective molecular volume (in the area x > 0). This, in turn, generates pressure difference (with respect to the area without field, x < 0) that creates a material (microparticle) flow in the +x direction (Fig. 10a). This movement generates a director reorientation wave (in the area x < 0) that propagates in the −x direction. At the same time, there is no such wave in the opposite direction (in the area x > 0) since the NLC molecules are under strong stabilizing electric field. In contrast, switching OFF the potential causes an increase in the volume (in the area x > 0) and another pulse of reorientation wave is generated that now propagates towards the right part of the LC cell, Fig. 10b.

Our polarizing microscope observations showed (Fig. 6c) that there is a small non uniformity in the thickness of the LC cell. This causes the reorientation wave to be non-uniform. We think that this could be one of the reasons why the movement of microparticles is not strictly parallel to the x axis. We also think that the transient small side-jump of those microparticles along the y axis (when the potential is switched ON and OFF) could be related to the director’s transient out-of-plane reorientation (Fig. 8).

The overall reorientation level here is rather significant since we see more than 1.5 oscillations of light transmission through the polarimetric system. Given the optical birefringence of the NLC used (${\rm{\Delta }}n\equiv {\rm{n}}\parallel -{{\rm{n}}}_{\perp }\approx 0.2$, with ordinary and extraordinary refractive index values, respectively, ${{\rm{n}}}_{\perp }=1.536\,{\rm{and}}\,{\rm{n}}\parallel =1.739$) and the thickness of the LC cell (L = 20 μm), we can estimate (with strong boundary conditions and a bow-shaped director profile, Fig. 10)^3^ the maximum reorientation angle of the director (at z = L/4) to be ≈ 42°. This is a significant transient perturbation of the orientational configuration of the NLC film.

We do not know yet the specific mechanism that insures the continued movement of microparticles (up to 30 minutes) at far distances after the director’s transient reorientation has disappeared (with still maintained voltage ON). We think that it could be similar to osmotic forces since the pressure gradients take more time (compared to the director reorientation wave) to be resorbed. This question is currently being investigated and the corresponding results will be reported as soon as possible.

In the meantime, based on our experimental observations, we can conclude that the non-local character (both in time and space) of quite moderate electrical potential can have spectacular long-distance impact on the behavior of self-aligned molecular films. Indeed, it is interesting to notice that we observe these phenomena already for relatively weak electric fields (2.5 × 10^4^ V/cm), compared to those (3 × 10^5^ V/cm and 1.5 × 10^6^ V/cm) used, respectively, in^27^ and^28^ to obtain significant changes of the order parameter. Most importantly, our electric fields are also noticeably smaller (by an order of magnitude) compared to electric fields circulating in biological membranes during the action potential’s propagation (from 0.5 × 10^5^ V/cm to 3 × 10^5^ V/cm, see, e.g.^26^). Despite this fact, as we have demonstrated, even such small electrical potentials were enough to dynamically generate significant long distance fast orientational waves and slow microparticle transport. Thus, we think that the same phenomena may also play an important role in biological membranes. In fact, the electric field’s influence on membranes was already analyzed, but only from the local interaction’s point of view (such as electro-striction^41^, reorientation of head groups^42^ or electroporation^43^). In contrast, while the phenomena, described in our work, are also dependent on the local dielectric torque, the resulting microparticle transport and molecular reorientation wave are strongly non-local and may dramatically influence the morphology of the membrane (and, consequently, the ionic transport processes in those membranes) at long distances.

Namely and first of all, the changes observed in the order parameter will affect the molecular diffusion processes since, as it is well known^1^, in contrast to ordinary (isotropic) liquids, the molecular diffusion in LC materials is strongly anisotropic^6^. Thus, in a bulk NLC, the ratio r_d_ of diffusion coefficients in the directions parallel (D_II_) and perpendicular (D_⊥_) to the director may be as much as r_d_ ≡ D_II_/D_⊥_ ≈ 1.6^6^. This anisotropy may be even more dramatic in Smectic LC materials (with multiple molecular layers, like the myelin), where the diffusion of carriers in parallel to molecular layers (the director can be perpendicular to those layers), may be significantly higher compared with the diffusion between molecular layers (r_d_^−1^ ≈ 5–7)^6^. Obviously, any destabilization of the above-mentioned molecular orientation order will proportionately change the r_d_, since the higher the order parameter *S* the stronger this anisotropy is. The transient reorientation of molecules (observed in our work just after switching ON and switching OFF the electric field) will certainly decrease this anisotropy.

**Figure 11:**
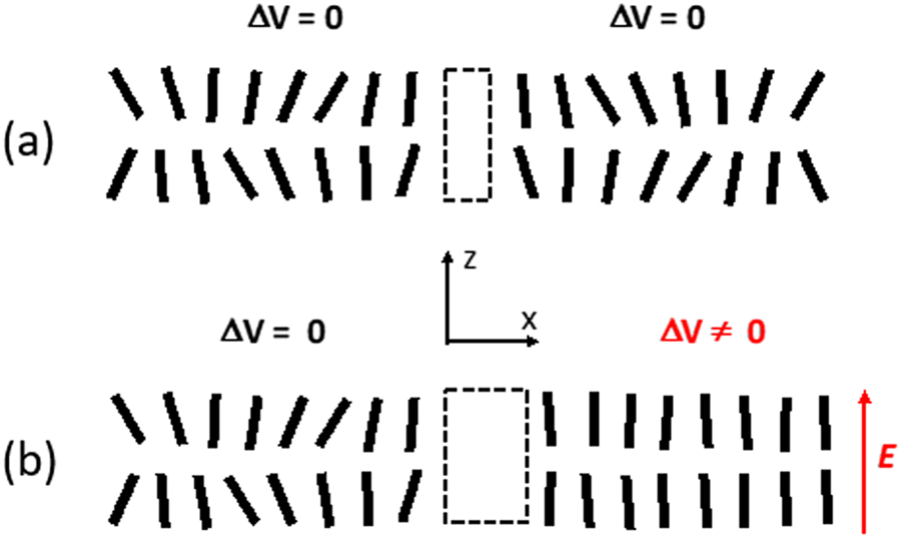
Schematic demonstration of the possible impact of the pressure gradient on cross-membrane channels (**a**) in the absence and (**b**) in the presence of a local electric potential pulse (on the right area).

Secondly, the local difference in pressure (around the area under transient electric potential) may also affect the performance of various ionic gating channels^26,44^. An example of such a channel is schematically shown (by the dashed rectangle in Fig. 11) that could be affected by the change of lateral pressure (along x axis, Fig. 11) and the transient passage of the reorientation wave (observed in our work).

It might be interesting to remind some well-known phases of action potential’s development in the cell membrane^26^). At the rest, the difference of the electric potential (across the membrane) may be at the order of ΔV = −75 mV. Thus, at this stage, the electric field is maximal across the membrane. Then, in the so-called “depolarization” phase, positively charged sodium ions Na+ quickly penetrate into the neuron through open voltage-gated sodium channels^26^, which reduces the absolute value of ΔV and then inverses its sign. If the mechanisms, revealed in our experiment, are present also in those systems, then such a transient change of ΔV will initiate rather strong material transport and molecular reorientation wave, which will propagate for very long distances (originating from the area where the electric potential was previously high) in the plane of the membrane. This reorientation wave will cause important morphological changes in the membrane, potentially affecting the boundary conditions on ionic channels (Fig. 11) and even influencing the triggering mechanisms for these channels.

There are several specificities that must be further taken into account. Our experiments were conducted by using AC square shaped voltage of 1 kHz frequency (this is the typical approach to avoid directional diffusion of ions in NLC materials^45^). However, the polarity of the electrical potential in the neural cell is also changing within few milliseconds. In addition, the control of the order parameter *S* can be achieved with both AC and/or DC electrical potentials (via the dielectric torque applied, respectively, to induced and permanent dipole moments) and, in this respect, all our observations (such as order parameter change, pressure gradient and orientation waves) are applicable to biological membranes too.

It is worth mentioning that the transport and reorientation phenomena have quite different time scales, but we should also keep in mind that the orientational response time of our system is conditioned by the thickness L of the NLC layer (see eq. 7) and it will be much shorter (τ ∝ L^2^) if we consider thinner molecular layers.

Furthermore, it is important to mention that our NLC layer was kept between two thick and solid substrates of the LC cell, which “contain and guide” the observed phenomena within a plane, while a biological membrane is a very flexible liquid bi-layer^46–48^. This flexibility probably would result in a local deformation of the entire membrane when the reorientation wave propagates along its surface. However, myelin sheath can limit this effect and, in this context, play the role of containing and guiding “substrates”. Thus, a fast inversion of the electrical potential in a given node of Ranvier (during the action potential propagation) would trigger the transient reorientation waves described above and material (e.g., ionic) transport along the axon. Once it arrived (guided) at the next Ranvier node, these perturbations might trigger significant changes in the properties of the membrane since it is no longer contained by the myelin. Thus, if this hypothesis is valid, the role of myelin sheath would not be limited to the “electric isolation” but also to the effective “guiding” of these reorientation waves and transport processes.

## Conclusions

In conclusion, our observations show that the local pulses of electrical potential can generate significant long-range morphological changes in self-oriented molecular films. This suggests that there must be corresponding perturbations in the cell membrane when its potential switches from −75 mV to +55 mV and then back to −75 mV. We think that these morphological changes may influence the dynamics of the action potential propagation by influencing the ion gating and by changing the diffusion and transport rates of ions along and across the membrane. The presence of myelin sheath will then “condition” the propagation of these excitation phenomena. Obviously, these predictions must be investigated in corresponding biological context to elucidate the impact of local electrical potential pulses on the operation of cells that we predict here.

### Ethical approval and informed consent

We confirm that we did not perform experiment on (i) live vertebrates (or higher invertebrates), (ii) humans or (iii) human samples.
